# Interactions between sleep disturbances and Alzheimer’s disease on brain function: a preliminary study combining the static and dynamic functional MRI

**DOI:** 10.1038/s41598-019-55452-9

**Published:** 2019-12-13

**Authors:** Kaicheng Li, Xiao Luo, Qingze Zeng, Yerfan Jiaerken, Shuyue Wang, Xiaopei Xu, Xiaojun Xu, Jingjing Xu, Chao Wang, Jiong Zhou, Peiyu Huang, Minming Zhang

**Affiliations:** 1 Department of Radiology, 2nd Affiliated Hospital of Zhejiang University School of Medicine, Zhejiang, China; 2 Department of Neurology, 2nd Affiliated Hospital of Zhejiang University School of Medicine, Zhejiang, China

**Keywords:** Sleep, Alzheimer's disease

## Abstract

Though sleep disturbance constitutes the risk factor for Alzheimer’s disease (AD), the underlying mechanism is still unclear. This study aims to explore the interaction between sleep disturbances and AD on brain function. We included 192 normal controls, 111 mild cognitive impairment (MCI), and 30 AD patients, with either poor or normal sleep (PS, NS, respectively). To explore the strength and stability of brain activity, we used static amplitude of low-frequency fluctuation (sALFF) and dynamic ALFF (dALFF) variance. Further, we examined white matter hyperintensities (WMH) and amyloid PET deposition, representing the vascular risk factor and AD-related hallmark, respectively. We observed that sleep disturbance significantly interacted with disease severity, exposing distinct effects on sALFF and dALFF variance. Interestingly, PS groups showed the dALFF variance trajectory of initially increased, then decreased and finally increased along the AD spectrum, while showing the opposite trajectory of sALFF. Further correlation analysis showed that the WMH burden correlates with dALFF variance in PS groups. Conclusively, our study suggested that sleep disturbance interacts with AD severity, expressing as effects of compensatory in MCI and de-compensatory in AD, respectively. Further, vascular impairment might act as important pathogenesis underlying the interaction effect between sleep and AD.

## Introduction

Alzheimer’s disease (AD) is the most common form of dementia, clinically characterized by progressive memory and other cognitive ability deficits. According to the 2018 World Alzheimer Report, there have been about 50 million AD patients worldwide, and this number will more than triple to 152 million by 2050^1^. Given that cure lifestyle factors might reduce or increase the risk of developing dementia, clinicians are paying more attention to modifying the reversible high-risk factors to delay the onset and progression of AD^2^. Recently, sleep disturbance was implicated as a risk factor for AD and has received much attention. About 25–40% of patients with AD or mild cognitive impairment (MCI) had abnormal sleep architecture^3–7^. Moreover, emerging evidence suggests that sleep disturbance may precede dementia onset and exacerbate cognitive symptoms^8,9^. Understanding the underlying neural mechanism of the sleep disturbance interacting with AD may shed light on future clinical interventions.

Fundamental research proposed that the pathological mechanism of interaction between sleep disturbance and AD may be the increased AD pathology deposit-amyloid beta (Aβ)^10,11^ or vascular impairments like the blood-brain barrier (BBB) dysfunction^12–14^. Such vascular risk factors may always appear as white matter hyperintensities (WMH) in neuroimage^15,16^. One previous study found that self-reported sleep-disordered breathing is associated with larger WMH volumes, suggesting that vascular impairments may mediate the sleep problem-related cognition decline^17^. Additionally, previous neuroimaging studies hinted that sleep disturbance might lead to the impairments of intrinsic brain activity, involving the hippocampal-neocortical, thalamo-cortical circuits, and further lead to cognitive dysfunction in normal subjects^18–24^. However, there is still a lack of *in vivo* study that aims to tackle the question of how sleep disturbances influence brain function in AD subjects.

Amplitude of low-frequency fluctuation (ALFF), as a high-sensitive brain measure reflecting the intrinsic brain activity, can be used to explore possible mechanisms. Specifically, the dynamic ALFF (dALFF) could reflect the temporal stability of the intrinsic brain activity^25^ while the static ALFF (sALFF) could reflect the strength of intrinsic brain activity. The increased temporal variance (decreased stability) or decreased temporal variance (excessive stability) may occur at the different pathological states and changed cognitive functions^26,27^. Previous studies have used the sALFF and dALFF variance in neuropsychiatric disorders, like AD and major depression disorder, and proved their effectiveness in reflecting the brain function and further clinical symptoms^28–30^. Thus, combining dynamic and static intrinsic brain activity information could help understand the neural mechanism underlying the interaction between sleep disturbances and dementia in different aspects^31,32^.

Taken together, the goal of our study aims to explore the interactions between sleep disturbances and AD severity on brain function *in vivo*. We used sALFF and dALFF to reflect the strength and stability of intrinsic brain activity, respectively. Moreover, we evaluated the WMH and amyloid PET deposit data to explore the possible mechanism.

## Methods and Materials

### Alzheimer’s disease neuroimaging and initiative

Data used in this study were obtained from the Alzheimer’s disease Neuroimaging Initiative (ADNI) database (http://adni.loni.usc.edu). The ADNI was initially launched in 2003 (ADNI-1) by the National Institute on Aging (NIA), the Food and Drug Administration (FDA), the National Institute of Biomedical Imaging and Bioengineering (NIBIB), and additional recruitment was made through ADNI-GO in 2009, ADNI-2 in 2010 and ADNI-3 in 2016. The primary goal of ADNI has been to identify serial magnetic resonance imaging (MRI), positron emission tomography (PET), biomarkers, and genetic characteristics that would support the early detection and tracking of AD, and improved clinical trial design. For up-to-date information, see http://www.adni-info.org. Similar information was described in a previous study^33^.

### Study participants

The ADNI project was approved by the Institutional Review Boards of all participating institutions, and all participants signed the informed written consent (http://adni.loni.usc.edu/wp-content/themes/freshnews-dev-v2/documents/policy/ADNI_Acknowledgement_List%205-29-18.pdf). We identified 192 normal controls (NC), 111 MCI subjects and 30 AD patients from the ADNI database (Supplementary Material 1, Flowchart). Each subject underwent 3D T1 weighted structural scan, resting-state functional MRI (rsfMRI), and comprehensive neuropsychological assessments.

We defined the NC as subjects had a Clinical Dementia Rating scale (CDR) score of 0, an Mini-Mental State Examination (MMSE) between 24 and 30 (inclusive), Wechsler Memory Scale Logical Memory, WMS-LM, delay recall performance ≥9 for subjects with 16 or more years of education; ≥5 for subjects with 8–15 years of education; and ≥3 for 0–7 years of education; non clinical depression (Geriatric Depression Scale-15, GDS-15 score < 6) and absence of dementia^34^. Regarding MCI inclusion criteria, subjects had preserved activities of daily living, non-dementia, and objective cognitive impairment as shown on the delayed recall test of the WMS-LM as well as a CDR score of 0.5^35^. As for AD, patients have MMSE of ≤26, CDR of ≥0.5, as well as met the NINCDS/ADRDA criteria for probable AD^36^. Exclusion criteria are listed below: (a) significant medical, neurological, and psychiatric illness; (b) obvious head trauma history; (c) use of non-AD-related medication known to influence cerebral function; (d) clinical depression; (e) alcohol or drug abuse. Similar criteria were described in previous studies^33,37^.

As an explorative investigation, we defined sleep state using medical history and night-time behavior scale in Neuropsychiatric Inventory (NPI) or a brief questionnaire form of NPI (NPI-Q), as previously described (Supplementary Material 2)^4,38–40^. Specifically, subjects with sleep disturbance (including sleep problems recorded in the medical history or abnormal NPI/NPI-Q (the scale score of the 9^th^ item: night-time behavioral disturbances ≥1)) were defined as the poor sleeper (PS); by contrast, subjects with both normal medical history and NPI/NPI-Q were defined as the normal sleeper (NS).

### Neuropsychological assessment

Each subjects finished comprehensive neuropsychological tests, including assessment of general mental status (MMSE) and other cognitive domains, involving episodic memory (Auditory Verbal Learning Test, AVLT; Wechsler Memory Scale Logical Memory, WMS-LM, immediate and delayed memory), attention (Trail-Making Test part A, TMT-A), visuospatial function (Clock-Drawing Test, CDT), decision-making function (Trail-Making Test part B, TMT-B), and language ability (Category verbal fluency).

### MRI acquisition and pre-processing

The structural images were obtained based on 3D Magnetization Prepared Rapid Acquisition Gradient Echo (MPRAGE) T1W sequence, with following parameters: voxel size = 1.1 × 1.1 × 1.2 mm^3^; echo time (TE) = 2.98 ms; inversion time (TI) = 900 ms; repetition time (TR) = 2300 ms; 170 sagittal slices; within plane FOV = 256 × 240 mm^2^. The FLAIR scans were obtained using an echo-planar imaging sequence: TE = 90 ms, TR = 9000 ms. Meanwhile, the rsfMRI images were obtained using an echo-planar imaging sequence: TE = 30 ms; TR = 3000 ms; the number of slices = 48; slice thickness = 3.3 mm; spatial resolution = 3.31 × 3.31 × 3.31 mm^3^. According to the ADNI scanning protocol, all subjects were instructed to open their eyes, focusing on a cross, and keep at rest calmly during the scan.

Neuroimaging data preprocessing was performed using the Data Processing Assistant and Resting-State fMRI (DPARSF; Yan and Zang; www.rfmri.org/DPASFA)^41^ based on the platform of Statistical Parametric Mapping 12 (SPM12; www.fil.ion.ucl.ac.uk/spm). The First 5 image volumes of rsfMRI scans were discarded for the signal equilibrium and subject’s adaptation to the scanning noise^41^. The remaining 135 images were corrected for timing differences and head motion (Friston 24 parameters)^42^. The image data with more than 3 mm maximum displacement in any of the x, y, or z directions or 3° of any angular motion were discarded. Then, T1-weighted images were co-registered to the mean rsfMRI image and spatially normalized to the Montreal Neurological Institute (MNI) standard space based on rigid-body transformation, then re-sampled into 3 mm × 3 mm × 3 mm cubic voxel. Finally, scrubbing was performed to reduce motion-related artifacts by using a framewise displacement (FD) threshold of 0.5, interpolating bad time points with cubic spline^43,44^. To control the residual effects of motion and other non-neuronal factors, we concluded covariates, including 24 head motion parameters and signals of white matter and cerebrospinal fluid (CSF)^41,42^.

### sALFF and dALFF variance calculation

The sALFF calculated the averaged ALFF across the scanning session and can be used to explore the strength of intrinsic brain activity. We calculated sALFF using the DPARSF software. Firstly, the time series were converted into the frequency domain using a fast Fourier transform at each voxel. Then, across 0.01–0.1 Hz, we computed and averaged the square root of the power spectrum. This averaged square root was taken as the sALFF at the given voxel^45^.

The dALFF was raised based on the theory that resting-state brain is a highly dynamic system on a variety of time scales^31,32,46–49^. Specifically, dALFF could distinguish the time course into several scales and calculate the variance value, to reflect the temporal stability of the intrinsic brain activity^25^. We calculated dALFF using the DynamicBC software (www.restfmri.net/forum/DynamicBC)^49^. The window size was set at 14 TR (42 s), and window step at 1TR based on previous studies, which suggested the window size in the range of 40 s to 100 s capturing brain dynamics reasonably^50,51^. The sliding-window was used for time-variant connectivity analysis. For each sliding window, the ALFF map was obtained. To study the temporal stability of intrinsic brain activity, we computed the dALFF variance. To test the reliability of the dALFF results, we repeatedly analyzed the dALFF with other window sizes (20, 26, 33 TR) (Supplementary Material 7).

### Assessment of WMH and amyloid deposit

To assess the cerebral vascular risk factor, we assessed the WMH burden. For each subject, the WMH lesion map was automatically created based on the T2 FLAIR image using Lesion Segmentation Toolbox in SPM12 (www.applied-statistics.de/lst)^52^. The masks were then manually corrected by two experienced neuroradiologists (MMZ, HPY). The WMH volume was subsequently calculated by multiplying voxel numbers by voxel size.

Regarding the amyloid PET, we acquired the composite Aβ deposition value (i.e., SUVR, standard uptake value ratio) from the ADNI database (UCBERKELEYAV45). To reflect the local amyloid deposition, we extracted the SUVRs based on the region of interest (ROIs). Notably, not all subjects had an amyloid PET scan. Thus 122 out of 176 NS and 145 out of 157 PS with amyloid PET data were included in the subsequent neuropathological correlation analysis.

### Statistical analysis

We used a Chi-squared test and analysis of variance (ANOVA) for categorical (gender) and continuous data (age, education), respectively (SPSS, version 19.0). Then, post-hoc analysis using a two-sample t-test was performed to reveal the source of ANOVA difference (significant at p < 0.05).

The interactions between sleep disturbance and disease severity on brain function (both sALFF and dALFF in a whole-brain voxel-wise way) were assessed with a 3 × 2 full factorial design, with groups (NC, MCI, and AD) and sleep state (NS and PS) as between-participant factors. Age, gender, education, and FD were employed as covariates (Supplementary Material 3, design matrix of the full factorial model). Firstly, F test was performed by setting the threshold at P < 0.001 at a voxel level, with P < 0.05 at the cluster level, corrected for multiple comparisons using the Gaussian random field (GRF) method^53–55^. Then, post-hoc analysis using a two-sample t-test was performed to explore the difference between groups by setting the threshold at P < 0.05. Moreover, the main effects of sleep quality, disease severity were showed in Supplementary Material 5. To explore the possible effect of GDS, we repeated our full factorial analysis by adding GDS as covariate (Supplementary Material 8).

Moreover, we investigated the associations between neuroimaging metrics (sALFF and dALFF variance in ROIs) and neuropathological as well as neuropsychological results using Pearson correlation analysis.

### Ethics approval and consent to participate

All procedures performed in studies involving human participants were in accordance by the ethical standards of the institutional and national research committee and with the 1964 Helsinki declaration and its later amendments or comparable ethical standards. Written informed consent was obtained from all participants and authorized representatives, and the study partners before any protocol-specific procedures were carried out in the ADNI study. More details in http://www.adni-info.org.

## Results

### Demographic and neuropsychological data

Finally, we defined six groups combining the disease severity (NC, MCI, AD) and sleep state (normal sleeper-NS, poor sleeper-PS): 123 NC with normal sleep (NC-NS); 69 NC with poor sleep (NC-PS); 39 MCI with normal sleep (MCI-NS); 72 MCI with poor sleep (MCI-PS); 14 AD with normal sleep (AD-NS); 16 AD with poor sleep (AD-PS).

We presented descriptive data as the mean ± standard deviation and percentage for continuous and dichotomous variables, respectively. All counterpart groups matched well for age and education and cognitive profile (Table 1 and Supplementary Material 4).

**Table 1:** Demographic and neuropsychological data.

Demographic characteristics	NC		MCI		AD		P-value	F-value
	NS (N = 123)	PS (N = 69)	NS (N = 39)	PS (N = 72)	NS (N = 14)	PS (N = 16)		
Age,y, mean (SD)	74.02 ± 7.13	74.77 ± 6.57	73.41 ± 8.07	73.78 ± 6.72	70.76 ± 8.24	74.66 ± 8.50	0.54	0.82
Female, n/N	70/123	46/69	19/39	30/72	5/14	9/16	0.04^g^	11.62
Education, y, mean (SD)	16.52 ± 2.16	16.39 ± 2.43	16.23 ± 3.17	16.10 ± 2.67	15.57 ± 2.77	16.38 ± 2.58	0.74	0.54
***Neuropsychiatric Scores***								
GDS	0.65 ± 0.98	1.20 ± 1.15	1.10 ± 1.27	1.94 ± 1.76	1.86 ± 0.95	1.75 ± 1.57	<0.001^abdfg^	10.79
***General mental status***								
MMSE	29.20 ± 1.06	29.12 ± 1.04	27.77 ± 2.64	27.03 ± 3.89	22.93 ± 2.67	20.81 ± 4.42	<0.001^defghi^	50.94
CDR global	0.00 ± 0.00	0.00 ± 0.00	0.42 ± 0.24	0.49 ± 0.32	0.75 ± 0.26	1.00 ± 0.55	<0.001^defghi^	127.96
CDR sum	0.01 ± 0.00	0.00 ± 0.00	1.21 ± 1.45	1.53 ± 2.07	3.79 ± 1.12	5.50 ± 3.46	<0.001^defghi^	72.06
***Memory function***								
WMS-LM immediate	14.39 ± 3.54	15.13 ± 3.17	9.74 ± 4.60	10.43 ± 4.39	4.25 ± 2.99	4.27 ± 2.99	<0.001^defghi^	46.90
WMS-LM delay	13.38 ± 3.73	14.03 ± 3.46	7.71 ± 5.59	8.10 ± 4.98	1.75 ± 2.22	1.07 ± 1.83	<0.001^defghi^	56.37
AVLT sum of trials 1–5	13.06 ± 2.11	13.36 ± 2.29	11.11 ± 3.63	10.42 ± 3.79	5.13 ± 3.36	4.13 ± 3.67	<0.001^defghi^	43.05
AVLT 30 min	7.77 ± 4.23	8.65 ± 3.54	4.82 ± 3.97	4.76 ± 5.57	0.50 ± 0.93	0.31 ± 1.25	<0.001^defghi^	19.08
***Attention***								
Log-transformed TMT-A	1.49 ± 0.13	1.46 ± 0.11	1.54 ± 0.15	1.56 ± 0.18	1.76 ± 0.24	1.86 ± 0.20	<0.001^efghi^	24.45
***Decision-making function***								
Log-transformed TMT-B	1.86 ± 0.20	1.83 ± 0.14	1.97 ± 0.22	1.96 ± 0.24	2.10 ± 0.18	2.35 ± 0.17	<0.001^cdfghi^	18.40
*Language*								
Category verbal fluency	21.57 ± 5.30	21.97 ± 4.70	18.44 ± 5.50	17.69 ± 6.14	10.50 ± 4.87	12.44 ± 5.81	<0.001^defghi^	18.06
***Visuospatial processing***								
CDT	4.75 ± 0.45	4.74 ± 0.53	4.32 ± 1.02	4.39 ± 1.00	3.14 ± 2.12	3.25 ± 1.57	<0.001^dghi^	15.19

Data are presented as means ± standard deviations.

Abbreviation: NC: normal control; MCI: mild cognitive impairment; AD: Alzheimer’s disease; NS: normal sleeper; PS: poor sleeper; GDS: Geriatric Depression Scale; MMSE, Mini-Mental State Examination; CDR: Clinical Dementia Rating; WMS-LM, Wechsler Memory Scale Logical Memory; AVLT, Auditory Verbal Learning Test; TMT, Trail-Making Test; CDT, Clock Drawing Test.

^a–i^Post-hoc analysis further revealed the source of ANOVA difference (^a^NC-NS vs NC-PS; ^b^MCI-NS vs MCI-PS; ^c^AD-NS vs AD-PS; ^d^NC-NS vs MCI-NS; ^e^MCI-NS vs AD-NS; ^f^NC-NS vs AD-NS; ^g^NC-PS vs MCI-PS; ^h^MCI-PS vs AD-PS; ^i^NC-PS vs AD-PS) (p < 0.05, significant difference between groups).

### sALFF results

**Figure 1:**
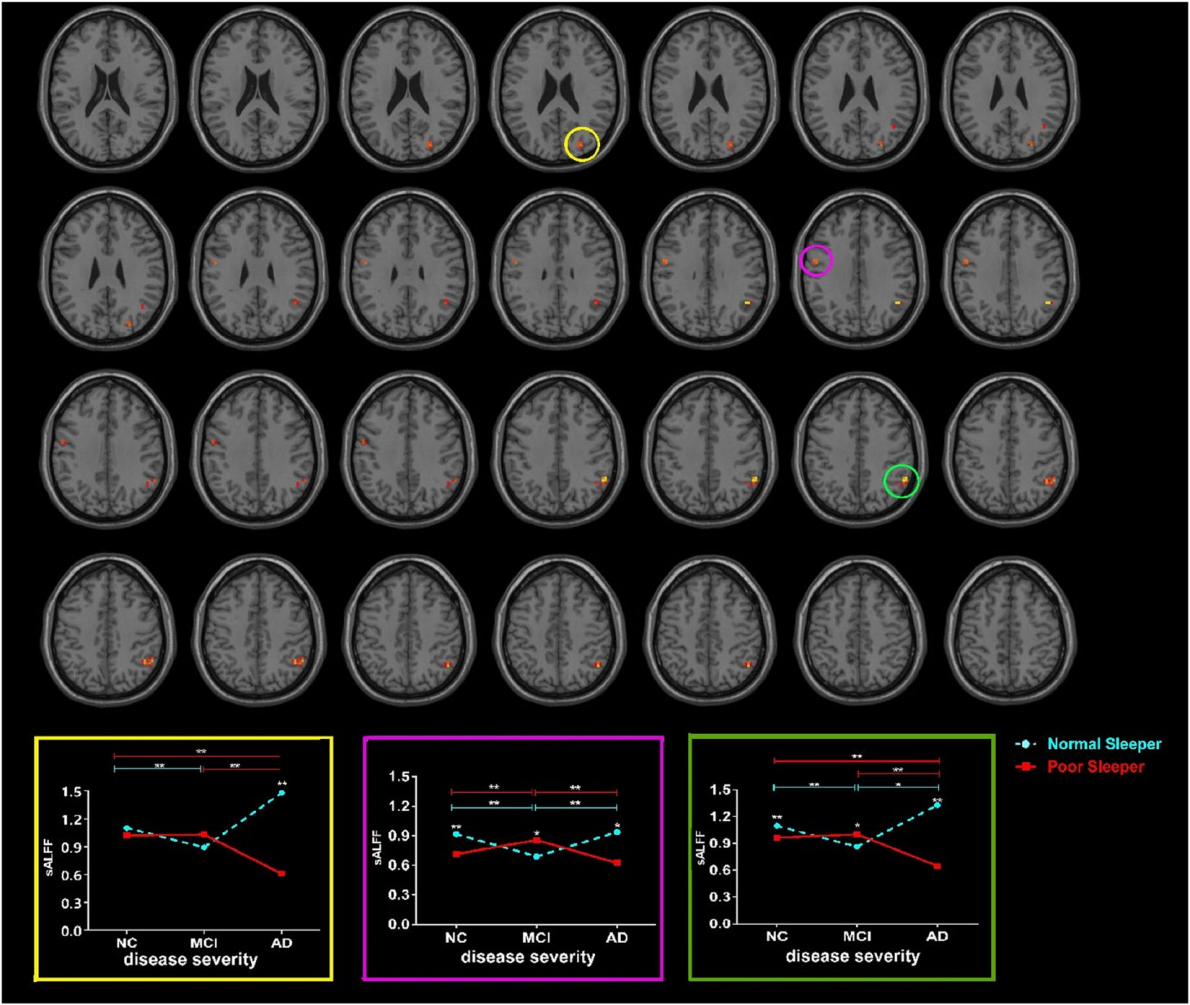
The sALFF differences between groups using F test by setting the threshold at voxel P < 0.001 and cluster P < 0.05, corrected by GRF. The interaction effects between sleep quality and disease severity were primarily located in the left precentral gyrus, right cuneus and right IPG (Full factorial model, voxel P < 0.001 and cluster P < 0.05, corrected by GRF, minimum cluster size is 8 voxels.). To be specific, NC-PS showed decreased sALFF in precentral gyrus and IPG when compared to NC-NS; MCI-PS showed increased sALFF in precentral gyrus and IPG when compared to MCI-NS; AD-PS showed decreased sALFF in precentral gyrus, IPG and cuneus when compared to AD-NS. Abbreviation: sALFF, static amplitude of low-frequency fluctuation; NC, normal control; MCI: mild cognitive impairment; AD: Alzheimer’s disease; NS: normal sleeper; PS: poor sleeper; IPG, inferior parietal region; GRF, Gaussian random field.

The interaction effects between sleep quality and disease severity were primarily located in the left precentral gyrus, right cuneus and right IPG (Fig. 1; Table 2: F test, threshold at voxel P < 0.001 and cluster P < 0.05, corrected by GRF; Tables 3 and 4: two-sample t-test, threshold at P < 0.05). To be specific, NC-PS had decreased sALFF in the precentral gyrus and IPG; MCI-PS had increased sALFF in the precentral gyrus and IPG; and AD-PS had decreased sALFF in the precentral gyrus, IPG, and cuneus when compared to their NS counterpart groups.

**Table 2:** The difference of sALFF and dALFF variance between groups using F test by setting the threshold at voxel P < 0.001 and cluster P < 0.05, corrected by GRF.

Neuroimaging Metrics	Cluster/peak regions	MNI coordinates			Extent	Max T
		X	Y	Z		
dALFF variance	Cerebelum_Crus1_R	45	−57	−30	10	8.53
	Cerebelum_8_R	24	−45	−45	10	9.45
	Insula_R	39	−3	6	10	10.68
	Insula_L	−39	−3	3	10	10.62
	Thalamus_L	−6	−6	0	9	9.38
	Temporal_Pole_Sup_L	−36	15	−30	14	14.27
	Hippocampal_R	21	−9	−21	8	10.74
	Rectus_R	6	27	−18	15	10.42
	SupraMarginal_R	57	−42	27	9	10.17
sALFF	Cuneus_R	27	−81	24	8	9.80
	Precentral_L	−51	−6	33	10	9.58
	Parietal_Inf_R	48	−54	33	35	12.49

Abbreviation: sALFF, static amplitude of low-frequency fluctuation; dALFF, dynamic amplitude of low-frequency fluctuation; GRF: Gaussian random field correction.

**Table 3:** Post-hoc analysis results within NC/MCI/AD subjects using two-sample t-test by setting the threshold at p < 0.05.

Post-hoc analysis	dALFF variance	sALFF
NC-PS > NC-NS	Cerebelum_8_R; Insula_R/L	No
NC-PS < NC-NS	NO	Parietal_Inf_R; Precentral_L
MCI-PS > MCI-NS	SupraMarginal_R	Parietal_Inf_R; Precentral_L
MCI-PS < MCI-NS	Insula_R/L; Temporal_Pole_Sup_L; Hippocampal_R	No
AD-PS > AD-NS	Cerebelum_8_R; Temporal_Pole_Sup_L; Rectus_R; Hippocampal_R	No
AD-PS < AD-NS	SupraMarginal_R	Precentral_L; Parietal_Inf_R; Cuneus

Abbreviation: NC: normal control; MCI: mild cognitive impairment; AD: Alzheimer’s disease; NS: normal sleeper; PS: poor sleeper; sALFF, static amplitude of low-frequency fluctuation; dALFF, dynamic amplitude of low-frequency fluctuation.

**Table 4:** Post-hoc analysis results between NC/MCI/AD subjects using two-sample t-test by setting the threshold at p < 0.05.

Post-hoc analysis	dALFF variance	sALFF
NC-PS < MCI-PS	Cerebelum_Crus1_R; Rectus_R; Temporal_Pole_Sup_L	Precentral_L
NC-PS > MCI-PS	No	No
MCI-PS < AD-PS	Cerebelum_8_R; Insula_R/L; Rectus_R; Temporal_Pole_Sup_L; Hippocampal_R; Thalamus_L	No
MCI-PS > AD-PS	No	Precentral_L; Parietal_Inf_R; Cuneus
NC-PS < AD-PS	Cerebelum_8_R; Insula_L; Rectus_R; Temporal_Pole_Sup_L; Hippocampal_R; Thalamus_L	No
NC-PS < AD-PS	Cerebelum_8_R; Insula_L; Rectus_R; Temporal_Pole_Sup_L; Hippocampal_R; Thalamus_L	No
NC-PS > AD-PS	No	Parietal_Inf_R; Cuneus

Abbreviation: NC: normal control; MCI: mild cognitive impairment; AD: Alzheimer’s disease; NS: normal sleeper; PS: poor sleeper; sALFF, static amplitude of low-frequency fluctuation; dALFF, dynamic amplitude of low-frequency fluctuation.

The main effect of sleep primarily located in the IPG, cerebellum, and motor-related region (Supplementary Material 5).

### dALFF variance results

**Figure 2:**
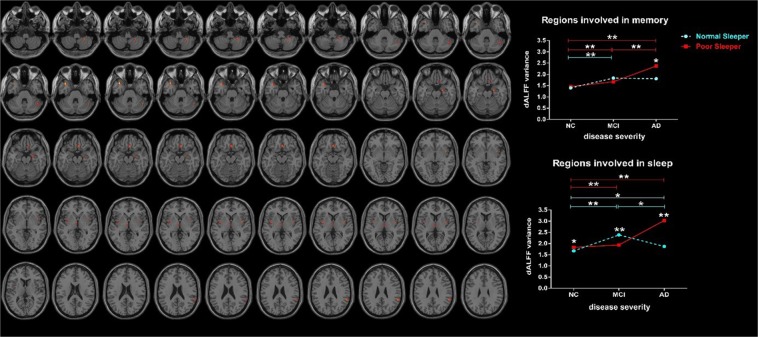
The dALFF variance differences between groups using F test by setting the threshold at voxel P < 0.001 and cluster P < 0.05, corrected by GRF. The interaction effects between sleep quality and disease severity were primarily located in regions involved in sleep and memory (Full factorial model, voxel P < 0.001 and cluster P < 0.05, corrected by GRF). To be specific, NC-PS showed increased dALFF variance in regions involved in sleep when compared to NC-NS. MCI-PS showed decreased dALFF variance in regions involved in sleep when compared to MCI-NS. AD-PS showed increased dALFF variance in both regions involved in sleep and memory when compared to AD-NS. Abbreviation: dALFF, dynamic amplitude of low-frequency fluctuation; NC, normal control; MCI: mild cognitive impairment; AD: Alzheimer’s disease; NS: normal sleeper; PS: poor sleeper; GRF, Gaussian random field.

The interaction effects between sleep quality and disease severity located in the insula, thalamus, cerebellum, superior temporal gyrus (STG), HP, gyrus rectus and supramarginal gyrus (Fig. 2, Table 2: F test, threshold at voxel P < 0.001 and cluster P < 0.05, corrected by GRF; Tables 3 and 4: two-sample t-test, threshold at P < 0.05). According to the previous studies about the hippocampus-dependent memories^56^ combined with the function of the brain region, we hypothetically classify these results into regions involved in sleep (insula, thalamus, cerebellum)^57–60^ and regions involved in memory (STG, HP, gyrus rectus and supramarginal gyrus)^61–64^. We noted that distributions of dALFF variance formed a biphasic trajectory along the AD spectrum. To be specific, relative to counterpart NS groups, NC-PS showed an increased dALFF variance in the regions involved in sleep; MCI-PS showed a decreased dALFF variance in the regions involved in sleep; while AD-PS showed an increased dALFF variance in both regions involved in sleep and memory. Moreover, we showed the functional change in each ROI in Supplementary Material 6.

The main effect of sleep primarily located in the cerebellum, gyrus rectus, and superior frontal gyrus (Supplementary Supplementary Material 5).

### Correlation analysis

**Figure 3:**
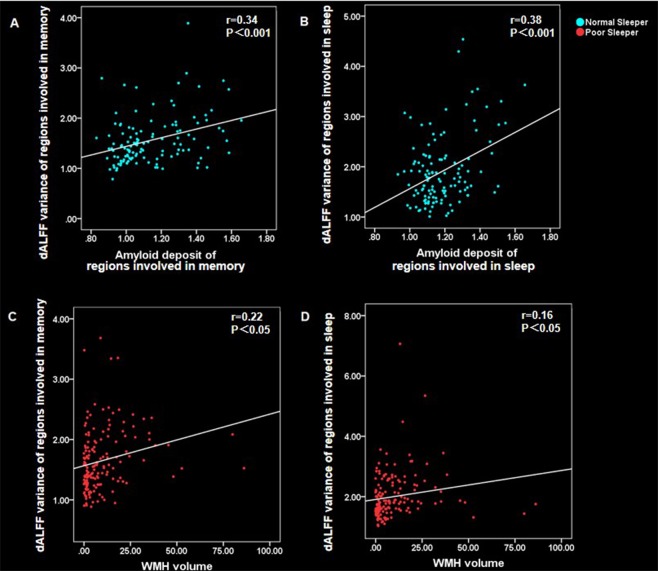
Scatter plot diagram of the correlation between ALFF values and WMH/amyloid. In subjects with normal sleep, the dALFF variance positively associated with amyloid deposit: (**A**) the dALFF variance of regions involved in memory was positively correlated with amyloid deposit (r = 0.34, p < 0.001); (**B**) the dALFF variance of regions involved in sleep was positively correlated with amyloid deposit (r = 0.38, p < 0.001). In subjects with poor sleep, the dALFF variance positively associated with WMH burden: (**C**) the dALFF variance of regions involved in memory was positively correlated with WMH burden (r = 0.22, P < 0.05); (**D**) the dALFF variance of regions involved in sleep was positively correlated with WMH burden (r = 0.16, P < 0.05). Abbreviation: dALFF, dynamic amplitude of low-frequency fluctuation; WMH, white matter hyperintensities.

To explore the possible mechanism underlying intrinsic brain activity alterations, we related ROI-derived ALFF values to the WMH and SUVR of amyloid PET in NS and PS separately. According to the Bonferroni correction criterion, the threshold was set at 0.0017 (p < 0.05/30). Based on this criterion, subjects with NS showed a significant association between dALFF variance and amyloid deposit. As for the subjects with PS, the dALFF variance showed a trend of positive association with WMH. To be specific, in subjects with PS, dALFF variance correlated with WMH in regions involved in memory (r = 0.22, P = 0.005) and sleep (r = 0.16, P = 0.041). In subjects with NS, dALFF variance correlated with amyloid deposit in regions involved in memory (r = 0.34, P = 0.00014) and sleep (r = 0.38, P = 0.000018) (Fig. 3).

**Figure 4:**
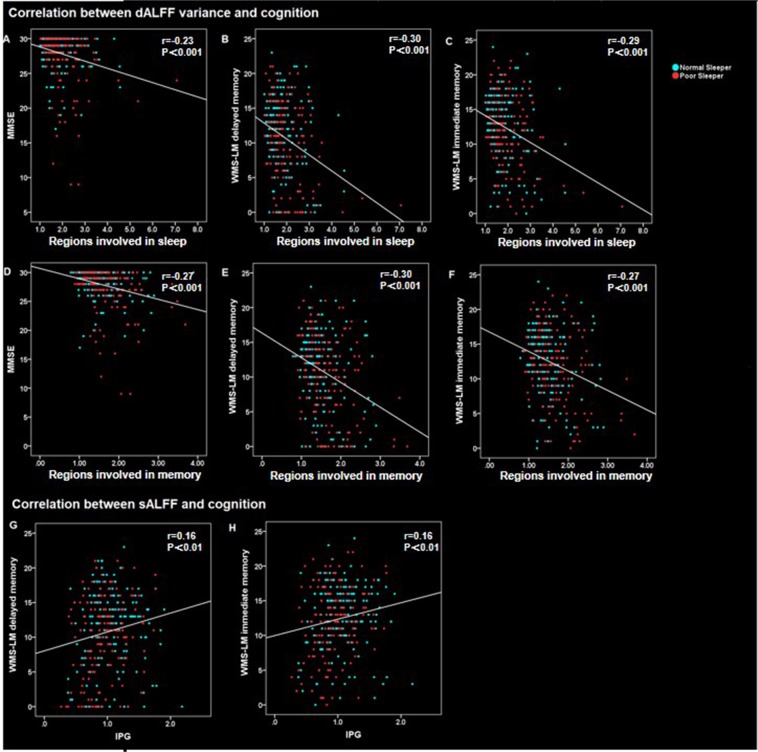
Scatter plot diagram of the correlation between ALFF values and cognition. Across groups, the dALFF variance negatively associated with cognition. (**A**) The dALFF variance of regions involved in sleep was negatively correlated with MMSE(r = −0.23, P < 0.001); (**B**) the dALFF variance of regions involved in sleep was negatively correlated with WMS-LM delayed memory (r = −0.30, P < 0.001); (**C**) the dALFF variance of regions involved in sleep was negatively correlated with WMS-LM immediate memory (r = −0.29, P < 0.001); (**D**) the dALFF variance of regions involved in memory was negatively correlated with MMSE (r = −0.27, P < 0.001); (**E**) the dALFF variance of regions involved in memory was negatively correlated with WMS-LM delayed memory (r = −0.30, P < 0.001); (**F**) the dALFF variance of regions involved in memory was negatively correlated with WMS-LM immediate memory (r = −0.27, P < 0.001). Across groups, we found a positive correlation between sALFF and cognition in IPG. (**G**) the sALFF of IPG was positively correlated with MS-LM delayed memory (r = 0.16, P < 0.05); (**H**) the sALFF of IPG was positively correlated with WMS-LM immediate memory (r = 0.16, P < 0.05). Abbreviation: dALFF, dynamic amplitude of low-frequency fluctuation; sALFF, static amplitude of low-frequency fluctuation; MMSE, Mini-Mental State Examination; WMS-LM, Wechsler memory scale-logical memory; IPG, inferior parietal gyrus.

Additionally, to prove the physiological signification of the ALFF, we correlated the functional changes to cognition. According to the Bonferroni correction criterion, the threshold should be set at 0.003 (p < 0.05/15). Based on this criterion, our results showed significant association between dALFF and cognition as well as a trend of a significant association between sALFF and cognition. To be specific, as for dALFF, we found a negative correlation between dALFF variance and cognition, in both regions involved in memory and sleep across groups. Detailed results were: dALFF variance of regions involved in memory and cognition (MMSE: r = −0.27, P = 4.72E-7; WMS-LM immediate memory: r = −0.27, P = 7.10E-7; MS-LM delayed memory: r = −0.30, P = 3.08E-8); dALFF variance of regions involved in sleep and cognition (MMSE: r = −0.23, P = 2.28E-5; WMS-LM immediate memory: r = −0.29, P = 2.14E-7; WMS-LM delayed memory: r = −0.30, P = 6.77E-8). As for sALFF analysis, we found a positive correlation between sALFF and cognition in IPG across groups (WMS-LM immediate memory: r = 0.16, P = 0.005; MS-LM delayed memory: r = 0.16, P = 0.005) (Fig. 4).

## Discussion

Our study explored the interactions between sleep disturbance and AD on intrinsic brain activity *in vivo*. We found that distributions of sALFF and dALFF variance formed a biphasic trajectory along the AD spectrum, mainly locating in the regions involved in memory and sleep. Further, our study suggested that vascular impairment might act as important pathogenesis underlying the interaction effect between sleep and AD.

### Stable intrinsic brain activity enables advanced cognitive function

The current study found that the dALFF variance negatively correlated with cognition, while sALFF positively correlated with cognition, including general cognition and memory. These results suggested that stable brain activity sustains the successful cognitive process. In this study, the interaction effect on the stability of intrinsic brain activity located in regions involved in memory (STG, HP, gyrus rectus, supramarginal gyrus)^61–64^ and sleep (insula, thalamus, cerebellum)^57–60^. These regions are involved in hippocampal-neocortical, thalamo-cortical, and cortico-thalamic circuits, which play an essential role in memory-consolidation during sleep^65–69^. Thereinto, the cerebellum participates in the sleep-wake cycle, and its malfunctions can lead to sleep disorders^70^. The thalamus selected information to be projected to the cortex while the cortex is asleep to protect sleep from external perturbations^71^, while the insula works to initiate and maintain sleep state^72^. On the other hand, HP plays a vital role in memory encoding and memory consolidation; STG is a substrate for auditory short-term memory^73^; gyrus rectus and supramarginal gyrus are also reported an association with memory loss in AD patients^61–64^. These regions interact with the cerebral cortex during sleep and contribute to the consolidation of memories^70,74–77^.

### Distributions of sALFF and dALFF variance formed a biphasic trajectory in subjects with poor sleep along the AD spectrum

At the stage of NC, NC-PS showed decreased stability of intrinsic brain activity in regions involved in sleep (especially the cerebellum and insula) compared to the NC-NS. This is in line with previous studies that reported that insula, thalamus, and cerebellum significantly contribute to sleep generation and stability^57–60^. Moreover, abnormal brain activity in thalamus and insula have also been reported in subjects with sleep problem^78,79^. As for the brain activity strength, NC-PS showed decreased functional strength in the precentral gyrus and IPG when compared to the NC-NS. Similarly, another study found sleep-related disrupted functional connectivity in IPG in older adults^80^. Moreover, evidence also comes from structural analysis, which found the association between poor sleep quality and reduced GM volume in the parietal regions and postcentral gyrus^72,81,82^. Conclusively, NC-PS showed both decreased stability and strength of intrinsic brain activity.

As the disease severity progresses, MCI-PS showed increased stability of intrinsic brain activity in regions involved in sleep when compared to the MCI-NS. We hypothesized that the increased functional stability is a compensatory enhancement, temporarily maintaining the brain function. The possible underlying mechanism may be the relatively severer pathological changes caused by sleep disturbance^10,11^. Such a compensatory hypothesis can partially be supported by the increased strength of intrinsic brain activity in the precentral gyrus and IPG in MCI-PS. Additionally, the well-matched cognitive profile between MCI-PS and MCI-NS also suggested the cognition maintain processes at the MCI stage.

Interestingly, MCI-PS also showed a trend of increased brain activity stability in regions involved in memory (especially the HP). Notably, HP is regarded as the critical structure in memory consolidation during sleep^56^ which involves information reactivation and hippocampo-neocortical transfer, based on the wide connection with other networks, including the hippocampo-striato-thalamo-cortical networks^83^, the hippocampus–amygdala system^84^, and hippocampal-cortical communication^85^. Accordingly, we speculated that the effect of sleep disturbance on brain activity in AD subjects might start from regions involved in sleep, then to the regions involved in memory, which may start from HP. Such impaired function will keep spreading as disease progression and ultimately lead to the irreversible cognition decline.

In the final disease stage, AD-PS showed decreased stability of intrinsic brain activity in both regions involved in sleep and memory when compared to AD-NS. The widespread functional alternations might reflect the de-compensation processes in the “biphasic model” in the AD spectrum. The decreased functional stability may suggest the overactivated functional oscillation and decreased synchronization, which leads to ineffective brain activity and finally causes cognition decline^86,87^. This decompensation hypothesis can also be proved by the decreased intrinsic brain activity strength in the precentral gyrus, IPG, and cuneus in AD-PS. Moreover, our analysis also found the relatively lower cognition in AD-PS, suggesting the severer cognition impairment.

Taken together, PS groups showed the dALFF variance trajectory of initially increased, then decreased and finally increased along the AD spectrum, while showing the opposite trajectory of sALFF. These changes may suggest the dynamic trajectory of an interaction effect between sleep disturbance and AD, expression as effects of compensatory in MCI and de-compensatory in AD, respectively.

### Vascular impairment might be an important pathogenesis underlying sleep-related impairments in AD

Different neural mechanisms may contribute to the opposite ALFF changes pattern in the NS and PS groups. In NS, we found the greater dALFF variance correlated with more amyloid deposition, rather than WMH burden, suggesting amyloid dominates the intrinsic brain activity. This is consistent with the amyloid hypothesis in previous AD studies. On the other hand, in PS, we found the greater dALFF variance correlated between severer WMH burden, rather than amyloid, indicating that vascular impairment works more prominently. This is partially in line with previous studies which reported that sleep might impair the function of BBB (including tight junction proteins loss and endothelial loss), which is a key pathway mediating the relationship between sleep disturbances and AD pathologies aggravation^12–14^. Similarly, previous studies also reported the link between sleep disturbance and vascular risk factors like subcortical infarcts and WMH, which may interfere with the sleep-wake cycle^88,89^.

Collectively, our results suggested the interaction effect between sleep disturbance and disease severity on brain function. These findings suggested that improving sleep quality^4,90^ or prevent cerebral vascular risk^91^ may achieve potentially better clinical prognosis for patients along the AD continuum.

### Limitation

There exist several limitations to our study. First, as an explorative investigation, our definition of sleep problem included insomnia, sleep apnea, and other self-reported information, which is somewhat heterogeneous. Further studies with sleep monitor equipment and standard quantitative sleep grades are urgent to verify our results. Moreover, our finding on NC is similar to previous studies, which suggested the feasibility of our result to some extent. Second, some amyloid PET data are missing (54 out of 176 NS and 2 out of 157 PS with amyloid PET data missing), which may reduce statistical power. Further studies with larger PET sample sizes are urgent. Thirdly, the sample size of the AD group is relatively small. Further studies with more AD subjects should be performed. Moreover, the observed effect might be affected by different sleep profiles during the scan since NS and PS may have a different risk to fall asleep in the scanner^92^. Further study should use the accurate eye-tracker system to reduce the possible effect. Finally, some medicine like acetylcholinesterase inhibitors may affect the sleep state. Further study should consider detailed medicine information to decrease the possible effect.

## Conclusion

Our study demonstrated that sleep disturbances interact with AD severity on static and dynamic intrinsic activity alteration, mainly locating in the regions involved in the memory and sleep. Moreover, the distributions of functional change are more likely to be a biphasic trajectory along the AD spectrum, with a compensatory enhancement in the MCI stage but decompensation in the AD stage. The mechanism underlying the interaction effects between sleep disturbances and AD may attribute to cerebral vascular-related risk factors.

## Supplementary information

Supplementary material

## Data Availability

The datasets generated and/or analysed during the current study are available in the ADNI study. More details in www.adni-info.org.
