# Supplementary material for: Interactions between sleep disturbances and Alzheimer’s disease on brain function: a preliminary study combining the static and dynamic functional MRI
Source: Sci Rep. 2019 Dec 13;9:19064. doi: 10.1038/s41598-019-55452-9 (PMC6911090; doi:10.1038/s41598-019-55452-9)
Supplement: Supplementary file 1 — Supplementary material [file 41598_2019_55452_MOESM1_ESM.pdf]

Interactions between sleep disturbances and Alzheimer's disease on brain function: a preliminary study combining the static and dynamic functional MRI

Kaicheng Li[1†], Xiao Luo[1†], Qingze Zeng[1], Yerfan Jiaerken[1], Shuyue Wang[1], Xiaopei Xu[1], Xiaojun Xu[1], Jingjing Xu[1], Chao Wang[1], Jiong Zhou[2], Peiyu Huang[*1], Minming Zhang[*1]

[1]Department of Radiology, 2nd Affiliated Hospital of Zhejiang University School of Medicine, China

[2]Department of Neurology, 2nd Affiliated Hospital of Zhejiang University School of Medicine, China

Corresponding to:

*Prof. Minming Zhang, MD, PhD

Department of Radiology, The 2nd Affiliated Hospital of Zhejiang University, School of Medicine, No.88 Jie-fang Road, Shang-cheng District, Hangzhou, China, 310009; Phone: 86-0571-87315255; Fax: 86-0571-87315255; Email address: zhangminming@zju.edu.cn

*Prof. Peiyu Huang, PhD

Department of Radiology, The 2nd Affiliated Hospital of Zhejiang University, School of Medicine, No.88 Jie-fang Road, Shang-cheng District, Hangzhou, China, 310009; Phone: 86-0571-87315255; Fax: 86-0571-87315255; Email address: hpyzju@foxmail.com

Supplementary Material 1. Flow chart of subjects inclusion

We identified 741 subjects who had sleep-related records in the medical history or night-time behavioral disturbance assessed by Neuropsychiatric Inventory (NPI) [1] or a brief questionnaire form of NPI (NPI-Q) [2] from ADNI GO, ADNI 2 and ADNI 3 database before 2018 September. After careful screening, 234 subjects were excluded due to rest-state

functional MRI (rsfMRI) data missing; 56 subjects were excluded due to inconformity between sleep complaint and rsfMRI time; 60 subjects were excluded due to different rsfMRI scan parameters or bad image quality; 58 subjects were excluded due to abnormal cognitive performance (Notably, in some samples, we selected data from the follow-up time points to ensure the time-correspondence of different image data, which may have different diagnosis from the baseline). Finally, we included 333 subjects undergone rsfMRI, structural scan, and comprehensive neuropsychological assessments. According to their cognition state, these subjects can be further grouped into normal control (NC), subjects with mild cognitive impairment (MCI) and Alzheimer's disease (AD) patients (Figure S1).

The inclusion criteria for NC as followed: (a) Mini-Mental State Examination (MMSE) between 24 and 30 (inclusive); (b) the clinical dementia rating (CDR) score of 0; (c) the normal Wechsler Memory Scale Logical Memory (WMS-LM), delay recall performance (in detail: ≥ 9 for subjects with 16 or more years of education; ≥ 5 for subjects with 8 – 15 years of education; and ≥ 3 for 0 – 7 years of education); (d) non-clinical depression (geriatric depression scale-15, GDS-15 score < 6) [3]; and (e) non-demented. The inclusion criteria for MCI subjects are: having (a) subjective memory complaints, either self-reported, reported by a study partner, or reported by a clinician; (b) decreased WMS-LM delayed recall performance; (c) CDR score of 0.5; (d) MMSE score between 24 and 30 (inclusive); and (e) general cognitive and functional performance sufficiently preserved such that a diagnosis of dementia could not be made by the site physician at the time of screening [4]. Regarding AD, the inclusion criteria were: (a) MMSE score of ≤ 26; (b) CDR ≥ 0.5, and (c) meeting the NINCDS/ADRDA criteria for probable AD [5].

We excluded subjects with the following manifestations: (a) significant medical, neurological, and psychiatric illness; (b) obvious head trauma history; (c) use of non-AD-related medication known to influence cerebral function; (d) clinical depression; (e) alcohol or drug abuse.

Figure S1. Flow chart of subjects inclusion

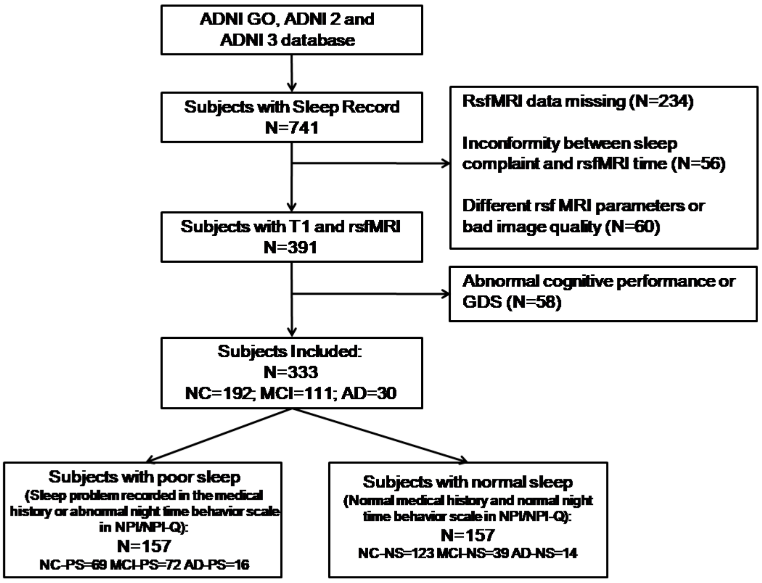

Abbreviation: rsfMRI: rest-state functional MRI; GDS: geriatric depression scale; NC: normal control; MCI: mild cognitive impairment; AD: Alzheimer's disease; NS: normal sleeper; PS: poor sleeper; NPI: Neuropsychiatric Inventory; NPI-Q: a brief questionnaire form of NPI.

Supplementary Material 2

We defined sleep quality using medical history (keywords: “insomnia”, “sleep problem”, and “sleep apnea”) and night-time behaviour scale in Neuropsychiatric Inventory (NPI) or a brief questionnaire form of NPI (NPI-Q), as previously described [2,6-8]. Specifically, we defined subjects with sleep disturbance (including sleep problems recorded in the medical history or abnormal NPI/NPI-Q) as the poor sleeper; by contrast, subjects with both normal medical history and NPI/NPI-Q were defined as the normal sleeper.

To be specific, NPI or NPI-Q [1,2] is a caregiver-based tool which assesses the possible presence of neuropsychiatric symptoms in dementia cases, including night-time behavioural disturbances. For detail, NPI/NPI-Q consists of 12 items (including delusions, hallucinations, agitation/aggression, dysphoria/depression, anxiety, euphoria/elation, apathy/indifference, disinhibition, irritability/lability, aberrant motor behaviors, night-time behavioral disturbances and appetite/eating disturbances) that the caregiver rates on a scale from 1 to 4 as to whether there has been no change or some degree of change and a severity range from 1 to 3 in each of neuropsychiatric symptoms.

Thereinto, the 9th item of NPI assesses the night-time behavior, which asked like "Does the patient awaken you during the night, rise too early in the morning, or take excessive naps during the day?" This item can reflect decreased nighttime sleep and increased daytime sleep, which both belong to the sleep disturbance and might impair cognition [9]. A sleep disturbance symptom was considered to be present if a caregiver reported the symptom (the scale score of the 9th item $\geqslant 1$). Previous studies have used NPI to measure sleep characteristics in MCI or AD and proved its validity [10-12].

Supplementary Material 3: Design matrix of the full factorial model

The interactions between sleep disturbance and disease severity on brain function (both sALFF and dALFF in a whole-brain voxel-wise way) were assessed with a 3 × 2 full factorial design, with groups (NC, MCI, and AD) and sleep state (NS and PS) as between-participant factors. Age, gender, education and FD were employed as covariates (Figure S2, S3).

Figure S2. Design matrix of the full factorial model

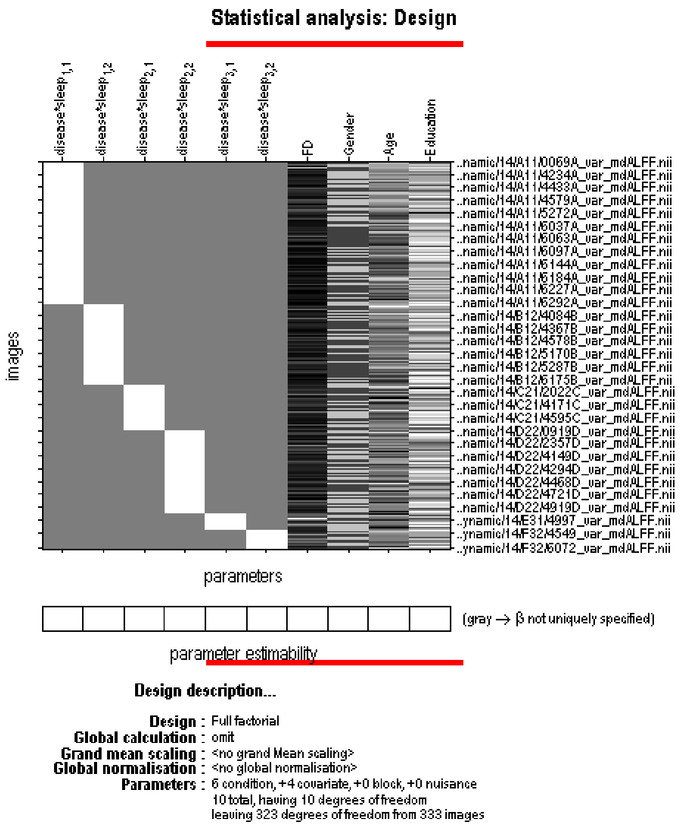

Supplementary Material 4: The main effect and interaction effect on neuropsychological performance

Table S1. The demographic and cognitive information of NC

**Demographic Characteristics**	**NC**		**P value**	**T/X^2**
	**NS (N=123)**	**PS (N=69)**		
**Age,y, mean (SD)**	**74.02±7.13**	**74.77±6.57**	**0.47**	**-0.72**
**Female, n (%)**	**70/123**	**46/69**	**0.22**	**1.76**
**Education,y, mean (SD)**	**16.52±2.16**	**16.39±2.43**	**0.70**	**0.38**

Neuropsychiatric Scores				
**GDS**	**0.65±0.98**	**1.20±1.15**	**0.001***	**-3.54**
General mental status				
**MMSE**	**29.20±1.06**	**29.12±1.04**	**0.59**	**0.55**
**CDR global**	**0.00±0.00**	**0.00±0.00**	**0.46**	**0.75**
**CDR sum**	**0.01±0.00**	**0.00±0.00**	**0.46**	**0.75**
Memory function				
**WMS-LM immediate**	**14.39±3.54**	**15.13±3.17**	**0.15**	**-1.45**
**WMS-LM delay**	**13.38±3.73**	**14.03±3.46**	**0.24**	**-1.18**
**AVLT sum of trials 1–5**	**13.06±2.11**	**13.36±2.29**	**0.36**	**-0.93**
**AVLT 30min**	**7.77±4.23**	**8.65±3.54**	**0.14**	**-1.47**
Attention				
**Log-transformed TMT-A**	**1.49±0.13**	**1.46±0.11**	**0.14**	**1.49**
Decision-making function				
**Log-transformed TMT-B**	**1.86±0.20**	**1.83±0.14**	**0.42**	**0.81**
Language				
**Category verbal fluency**	**21.57±5.30**	**21.97±4.70**	**0.60**	**-0.53**
Visuospatial processing				
**CDT**	**4.75±0.45**	**4.74±0.53**	**0.84**	**0.21**

Data are presented as means ± standard deviations.

Abbreviation: NC: normal control; NS: normal sleeper; PS: poor sleeper; GDS: Geriatric Depression Scale; MMSE, Mini-Mental State Examination; CDR: Clinical Dementia Rating; WMS-LM, Wechsler Memory Scale

Logical Memory; AVLT, Auditory Verbal Learning Test; TMT, Trail-Making Test; CDT, Clock Drawing Test.

*p<0.05, significant difference between groups

Table S2. The demographic and cognitive information of MCI

**Demographic characteristics**	**MCI**		**P value**	**T/X^2**
	**NS (N=39)**	**PS (N=72)**		
**Age,y, mean (SD)**	**73.41±8.07**	**73.78±6.72**	**0.80**	**-0.25**
**Female, n (%)**	**19/39**	**30/72**	**0.52**	**1.30**
**Education,y, mean (SD)**	**16.23±3.17**	**16.10±2.67**	**0.81**	**0.24**
Neuropsychiatric Scores				
**GDS**	**1.10±1.27**	**1.94±1.76**	**0.01***	**-2.64**
General mental status				
**MMSE**	**27.77±2.64**	**27.03±3.89**	**0.29**	**1.07**
**CDR global**	**0.42±0.24**	**0.49±0.32**	**0.24**	**-1.19**
**CDR sum**	**1.21±1.45**	**1.53±2.07**	**0.39**	**-1.29**
Memory function				
**WMS-LM immediate**	**9.74±4.60**	**10.43±4.39**	**0.46**	**-0.75**
**WMS-LM delay**	**7.71±5.59**	**8.10±4.98**	**0.72**	**-0.36**
**AVLT sum of trials 1–5**	**11.11±3.63**	**10.42±3.79**	**0.37**	**0.91**
**AVLT 30min**	**4.82±3.97**	**4.76±5.57**	**0.95**	**0.06**
Attention				

**Log-transformed TMT-A**	**1.54±0.15**	**1.56±0.18**	**0.50**	**-0.67**
Decision-making function				
**Log-transformed TMT-B**	**1.97±0.22**	**1.96±0.24**	**0.79**	**0.27**
Language				
**Category verbal fluency**	**18.44±5.50**	**17.69±6.14**	**0.53**	**0.63**
Visuospatial processing				
**CDT**	**4.32±1.02**	**4.39±1.00**	**0.72**	**-0.36**

Data are presented as means ± standard deviations.

Abbreviation: MCI: mild cognitive impairment; NS: normal sleeper; PS: poor sleeper; GDS: Geriatric Depression Scale; MMSE, Mini-Mental State Examination; CDR: Clinical Dementia Rating; WMS-LM, Wechsler Memory Scale Logical Memory; AVLT, Auditory Verbal Learning Test; TMT, Trail-Making Test; CDT, Clock Drawing Test.

*p<0.05, significant difference between groups

Table S3. The demographic and cognitive information of AD

**Demographic characteristics**	**AD**		**P value**	**T/X^2**
	**NS (N=14)**	**PS (N=16)**		
**Age,y, mean (SD)**	**70.76±8.24**	**74.66±8.50**	**0.21**	**-1.27**
**Female, n(%)**	**5/14**	**9/16**	**0.30**	**1.27**
**Education,y, mean (SD)**	**15.57±2.77**	**16.38±2.58**	**0.42**	**-0.82**
Neuropsychiatric Scores				
**GDS**	**1.86±0.95**	**1.75±1.57**	**0.83**	**0.22**
General mental status				

**MMSE**	**22.93±2.67**	**20.81±4.42**	**0.13**	**1.56**
**CDR global**	**0.75±0.26**	**1.00±0.55**	**0.13**	**-1.56**
**CDR sum**	**3.79±1.12**	**5.50±3.46**	**0.09**	**-1.77**
Memory function				
**WMS-LM immediate**	**4.25±2.99**	**4.27±2.99**	**0.99**	**-0.02**
**WMS-LM delay**	**1.75±2.22**	**1.07±1.83**	**0.39**	**0.88**
**AVLT sum of trials 1–5**	**5.13±3.36**	**4.13±3.67**	**0.53**	**0.65**
**AVLT 30min**	**0.50±0.93**	**0.31±1.25**	**0.71**	**0.37**
Attention				
**Log-transformed TMT-A**	**1.76±0.24**	**1.86±0.20**	**0.26**	**-1.15**
Decision-making function				
**Log-transformed TMT-B**	**2.10±0.18**	**2.35±0.17**	**0.02***	**-2.57**
Language				
**Category verbal fluency**	**10.50±4.87**	**12.44±5.81**	**0.43**	**-0.81**
Visuospatial processing				
**CDT**	**3.14±2.12**	**3.25±1.57**	**0.89**	**-0.14**

Data are presented as means ± standard deviations.

Abbreviation: AD: Alzheimer's disease; NS: normal sleeper; PS: poor sleeper; GDS: Geriatric Depression Scale; MMSE, Mini-Mental State Examination; CDR: Clinical Dementia Rating; WMS-LM, Wechsler Memory Scale Logical Memory; AVLT, Auditory Verbal Learning Test; TMT, Trail-Making Test; CDT, Clock Drawing Test.

*p<0.05, significant difference between groups

Table S4. The main effect and interaction effect on neuropsychological performance

**Demographic**	**NC**	**MCI**	**AD**			**P-value**

**characteristics**	**NS (N=123)**	**PS (N=69)**	**NS (N=39)**	**PS (N=72)**	**NS (N=14)**	**PS (N=16)**	**P-value** Sleep quality	**P-value** Disease severity	Sleep × Disease interaction
**MMSE**	29.20±1.06	29.12±1.04	27.77±2.64	27.03±3.89	22.93±2.67	20.81±4.42	0.01	＜0.001	0.10
**WMS-LM immediate**	14.39±3.54	15.13±3.17	9.74±4.60	10.43±4.39	4.25±2.99	4.27±2.99	0.48	＜0.001	0.75
**WMS-LM delay**	13.38±3.73	14.03±3.46	7.71±5.59	8.10±4.98	1.75±2.22	1.07±1.83	0.80	＜0.001	0.65
**AVLT sum of trials 1–5**	13.06±2.11	13.36±2.29	11.11±3.63	10.42±3.79	5.13±3.36	4.13±3.67	0.41	＜0.001	0.31
**AVLT 30min**	7.77±4.23	8.65±3.54	4.82±3.97	4.76±5.57	0.50±0.93	0.31±1.25	0.96	＜0.001	0.55
**Log-transformed TMT-A**	1.49±0.13	1.46±0.11	1.54±0.15	1.56±0.18	1.76±0.24	1.86±0.20	0.14	＜0.001	0.06
**Log-transformed TMT-B**	1.86±0.20	1.83±0.14	1.97±0.22	1.96±0.24	2.10±0.18	2.35±0.17	0.12	＜0.001	0.07
**Category verbal fluency**	21.57±5.30	21.97±4.70	18.44±5.50	17.69±6.14	10.50±4.87	12.44±5.81	0.42	＜0.001	0.44
**CDT**	4.75±0.45	4.74±0.53	4.32±1.02	4.39±1.00	3.14±2.12	3.25±1.57	0.87	＜0.001	0.87

Data are presented as means ± standard deviations.

Abbreviation: AD: Alzheimer's disease; NS: normal sleeper; PS: poor sleeper; GDS: Geriatric Depression Scale; MMSE, Mini-Mental State Examination WMS-LM, Wechsler Memory Scale Logical Memory; AVLT, Auditory Verbal Learning Test; TMT, Trail-Making Test; CDT, Clock Drawing Test.

Supplementary Material 5: The main effects of disease and sleep

The main effect of disease

As for the static amplitude of low-frequency fluctuation (sALFF) analysis, the main effect of disease was widely observed, especially in the temporal region (Table S5, Figure S3).

As for the dynamic ALFF (dALFF) variance analysis, the main effect of disease was widely observed in the cerebellum, temporal region and parietal region (Table S5, Figure S4). The findings are in line with previous studies on the anatomical location of AD-related changes.

Table S5. The main effect of disease

**Neuroimaging Metrics**	**Cluster/peak regions**	**MNI coordinates**			**Extent**	**Max T**
		**X**	**Y**	**Z**		
sALFF	**Cerebelum_9_R**	**9**	**-42**	**-51**	**14**	**11.73**
	**Cerebelum_9_L**	**-12**	**-45**	**-51**	**14**	**11.40**
	**Cerebelum_8_R**	**36**	**-54**	**-45**	**11**	**12.16**
	**Temporal_Pole_Sup_R**	**36**	**12**	**-30**	**10**	**9.15**
	**Temporal_Pole_Sup_L**	**-36**	**9**	**-27**	**62**	**15.64**
	**Amygdala_R**	**24**	**0**	**-21**	**14**	**11.33**
	**Temporal_Inf_L**	**-42**	**-36**	**-18**	**17**	**18.27**
	**Temporal_Inf_R**	**48**	**-27**	**-27**	**12**	**11.98**
	**Fusiform_L**	**-33**	**-54**	**-6**	**19**	**17.29**
	**Insula_L**	**-33**	**18**	**12**	**10**	**12.74**
	**SupraMarginal_R**	**60**	**-45**	**24**	**10**	**10.17**
	**Calcarine_R**	**18**	**-57**	**6**	**11**	**13.21**
	**Frontal_Mid_R**	**42**	**6**	**39**	**14**	**10.54**
	**Temporal_Sup_R**	**48**	**-24**	**9**	**11**	**10.84**
	**Cerebelum_8_L**	**-30**	**-60**	**-54**	**14**	**10.40**
	**Cerebelum_7b_L**	**-36**	**-48**	**-42**	**18**	**15.79**

dALFF variance	Cerebelum_Crus1_L	-42	-60	-27	27	12.00
	Cerebelum_6_R	36	-48	-27	71	15.93
	Vermis_4_5	6	-51	3	17	14.03
	Fusiform_L	-36	-15	-30	23	13.54
	Temporal_Inf_L	-42	-39	-18	80	16.92
	Temporal_Inf_R	50	-9	-36	31	13.06
	Temporal_Pole_Sup_L	-36	9	-27	155	18.35
	Temporal_Pole_Sup_R	36	18	-30	20	15.27
	Frontal_Mid_L	-30	39	45	33	12.16
	Frontal_Mid_L	-39	15	54	24	14.27
	Angular_R	42	-57	24	18	13.44
	Parietal_Sup_L	-15	-66	48	20	13.71
	Parietal_Inf_L	-24	-54	54	14	14.56

Abbreviation: sALFF, static amplitude of low-frequency fluctuation; dALFF, dynamic amplitude of low-frequency fluctuation.

Figure S3. The main effect of disease on sALFF

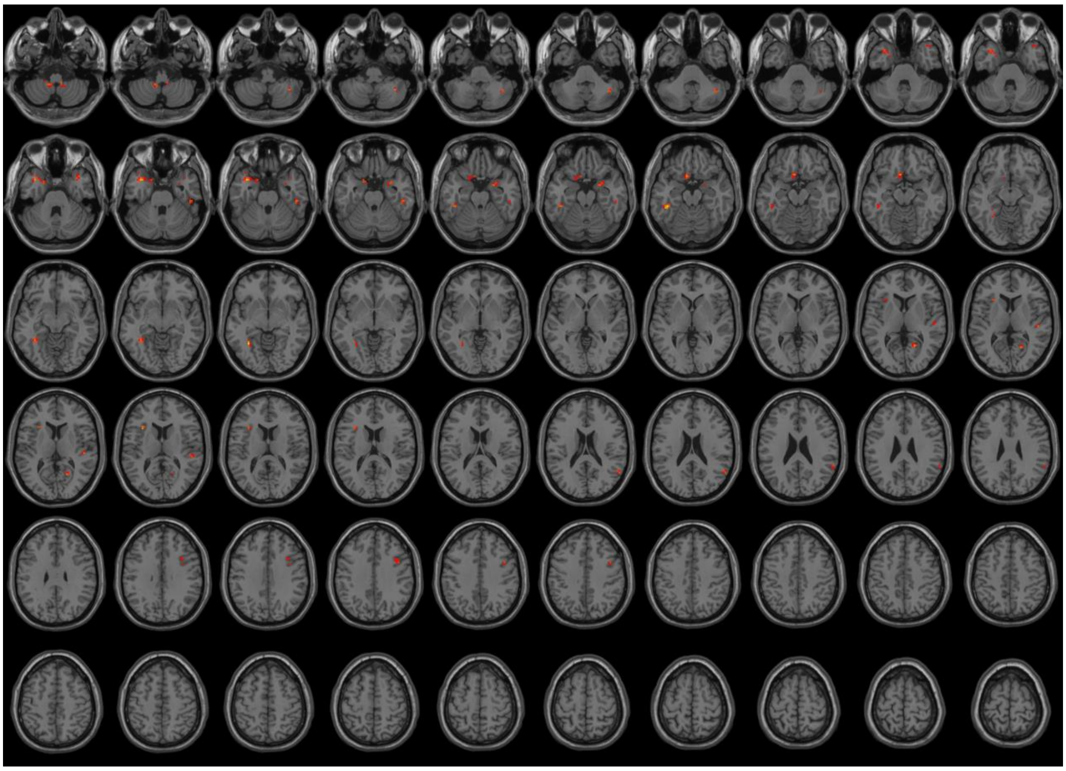

Abbreviation: sALFF, static amplitude of low-frequency fluctuation

Figure S4. The main effect of disease on dALFF variance

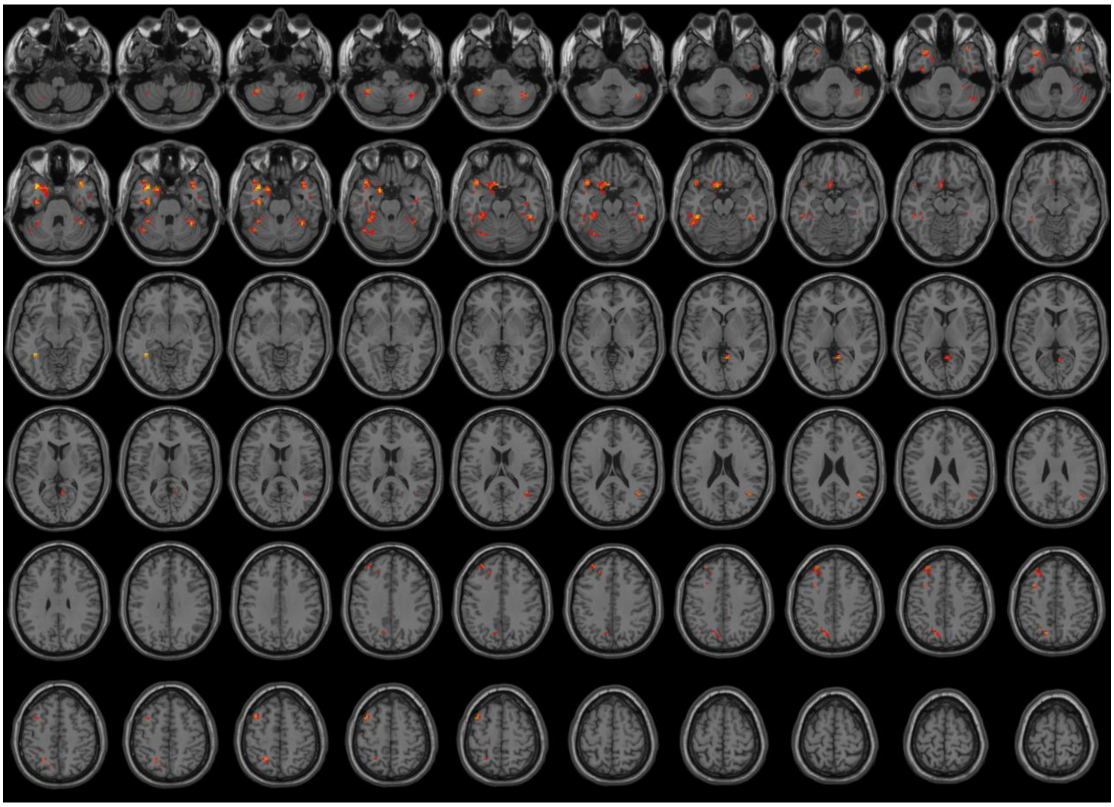

Abbreviation: dALFF, dynamic amplitude of low-frequency fluctuation.

The main effect of sleep

As for the sALFF analysis, the main effect of sleep on the ALFF was primarily located in IPG, cerebellum, and motor-related region (Table S6, Ficture S5). As for the dALFF variance analysis, the main effect of sleep quality on the ALFF was primarily located in the cerebellum, gyrus rectus and superior frontal gyrus (Table S6, Figure S6). The findings are in line with previous studies reporting sleep relates with the motor memory consolidation and emotional memory during sleep. This study suggested it widely involved in networks including hippocampo-striato-thalamo-cortical networks [13], hippocampus–amygdala system [14] and hippocampal-cortical communication [15]. These findings are partially similar to previous studies which reported that poor sleep quality was associated with reduced GM volume in the frontal cortex, temporal, parietal regions, postcentral gyrus, precuneus and insula [16-18].

Table S6. The main effect of sleep

**Neuroimaging Metrics**	**Cluster/peak regions**	**MNI coordinates**			**Extent**	**Max T**
		**X**	**Y**	**Z**		
**sALFF**	**Cerebelum_Crus1_R**	**45**	**-51**	**-36**	**8**	**17.80**
	**Temporal_Inf_R**	**45**	**-54**	**-9**	**9**	**21.66**
	**Occipital_Sup_R**	**24**	**-78**	**24**	**10**	**21.84**
	**Precuneus_L**	**-18**	**-63**	**36**	**11**	**17.55**
	**Parietal_Inf_L**	**-54**	**-30**	**42**	**8**	**18.51**
	**Parietal_Inf_R**	**54**	**-48**	**39**	**33**	**26.40**
	**Postcentral_L**	**-51**	**-6**	**42**	**8**	**16.09**

	**Postcentral_R**	**30**	**-42**	**54**	**9**	**17.15**
	**Supp_Motor_Area_R**	**12**	**15**	**51**	**9**	**16.31**
	**Supp_Motor_Area_L**	**-6**	**9**	**57**	**7**	**15.63**
**dALFF variance**	**Cerebelum_8_R**	**30**	**-42**	**-51**	**12**	**18.77**
	**Cerebelum_Crus2_R**	**45**	**-69**	**-45**	**16**	**18.21**
	**Cerebelum_Crus1_R**	**45**	**-51**	**-36**	**8**	**16.24**
	**Cerebelum_3_R**	**9**	**-36**	**-18**	**9**	**13.80**
	**Frontal_Sup_Orb_L**	**-15**	**27**	**-21**	**9**	**17.84**
	**Rectus_R**	**3**	**27**	**-15**	**34**	**21.88**

Abbreviation: sALFF, static amplitude of low-frequency fluctuation; dALFF, dynamic amplitude of low-frequency fluctuation.

Figure S5. The main effect of sleep on sALFF

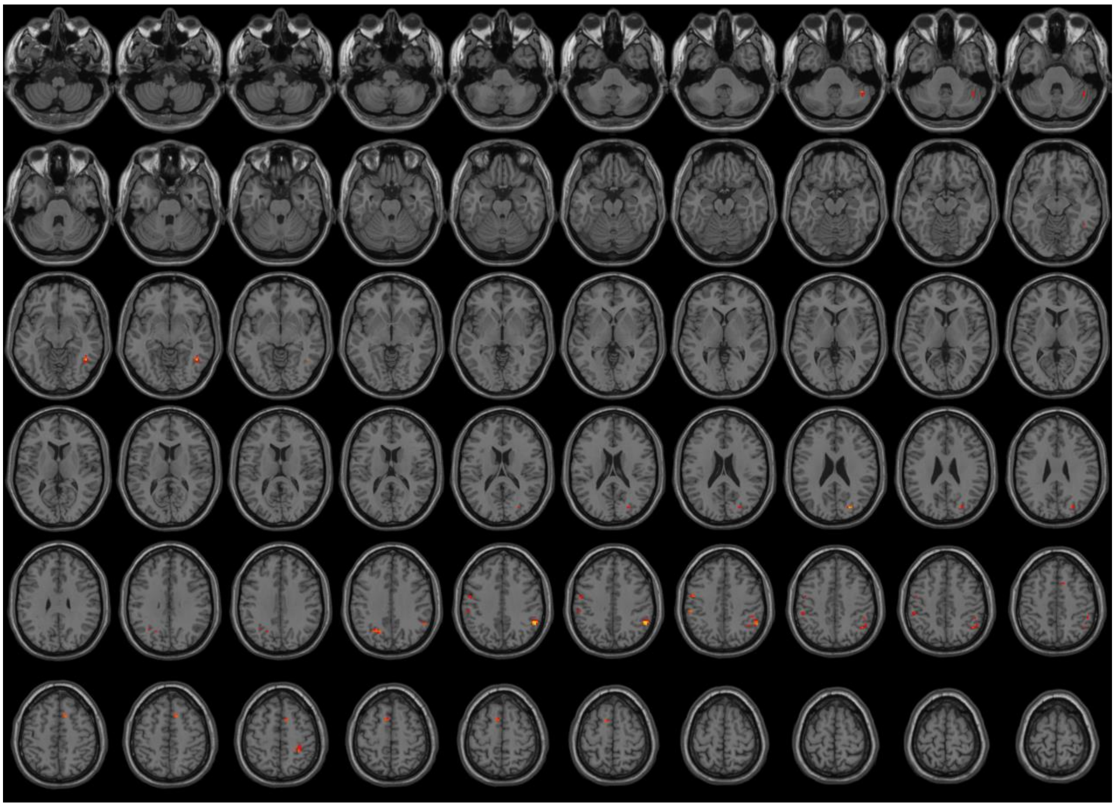

Abbreviation: sALFF, static amplitude of low-frequency fluctuation

Figure S6. The main effect of sleep on dALFF variance

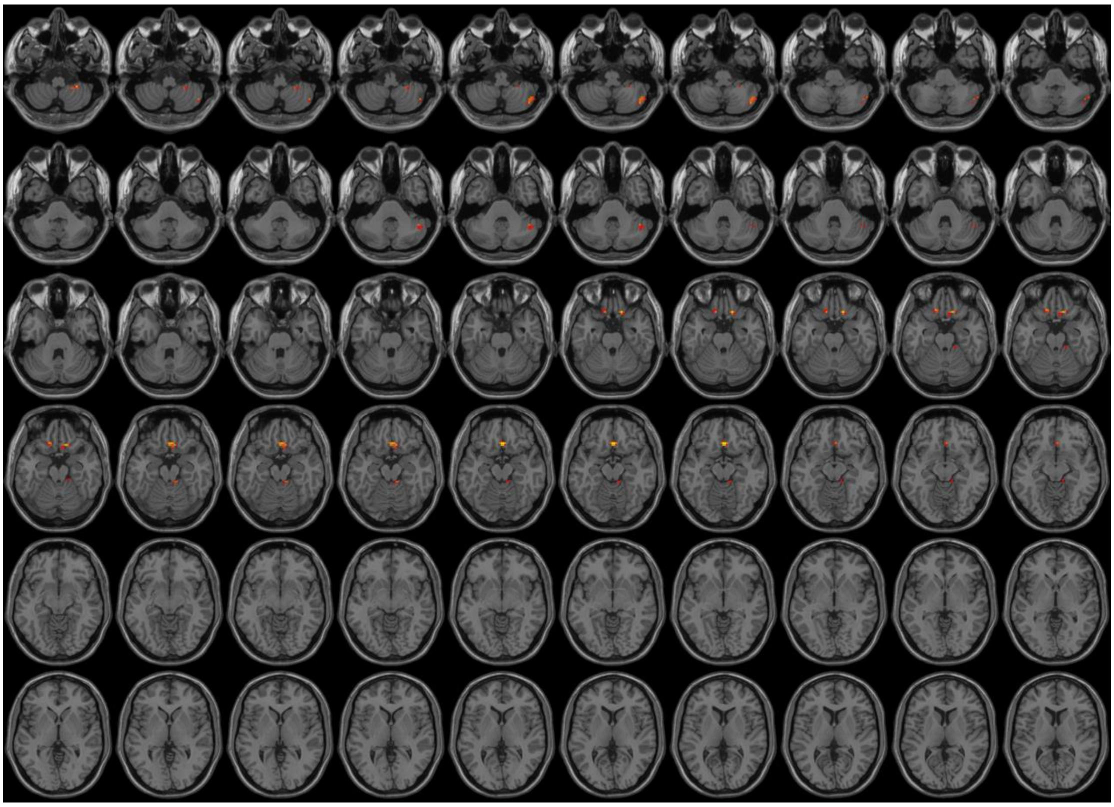

Abbreviation: dALFF, dynamic amplitude of low-frequency fluctuation.

Supplementary Material 6: The interaction effects between sleep quality and disease severity in every ROI with the window size of 14 TR

Here, we found the altered dALFF variance in superior temporal gyrus, hippocampus, gyrus rectus, supramarginal gyrus, insula, thalamus, and cerebellum. In subjects with poor sleep, we found an increased trend of dALFF variance along AD spectrum which suggested the decreased brain activity stability, especially involving the MCI to AD stage. As for the subjects with normal sleep, we found initially increased and then decreased dALFF variance along AD spectrum which may reflect the compensatory enhancement in the MCI stage and

decompensation in the AD stage (Figure S7).

Figure S7.

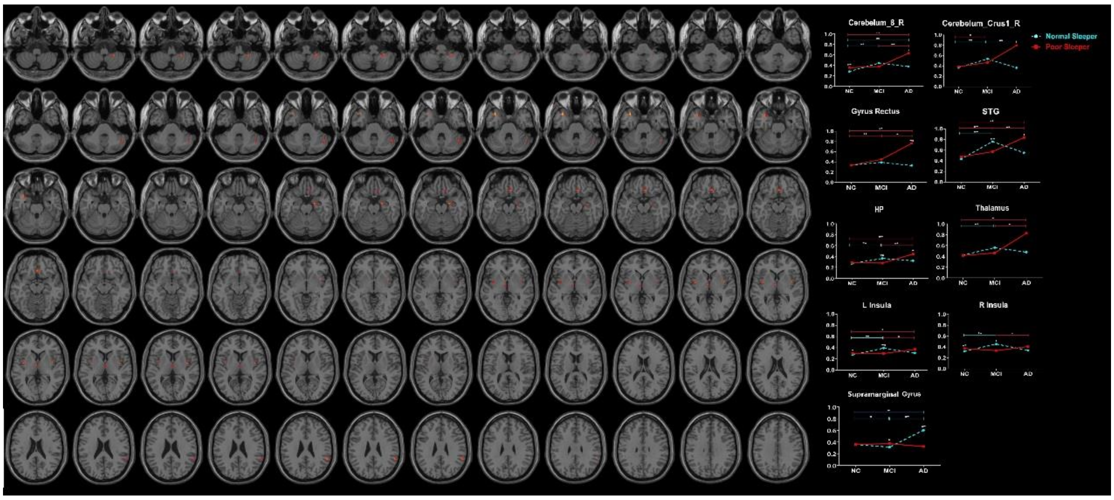

Abbreviation: STG, superior temporal gyrus; HP, hippocampus.

Supplementary Material 7: The interaction effects between sleep quality and disease severity with other window sizes

To test the reliability of the dALFF results, we also examined the results with window sizes of 20, 26, 33 TR (Table S7). The results are similar with the results under widow size of 14 TR, involving the cerebellum, temporal region, and insula.

Table S7. The interaction effects between sleep quality and disease severity with other window sizes

**Window sizes**	**Cluster/peak regions**	**MNI coordinates**			**Extent**	**Max T**
		**X**	**Y**	**Z**		
	**Cerebelum_8_R**	**27**	**-48**	**-48**	**9**	**9.60**
	**Cerebelum_Crus1_R**	**51**	**-57**	**-33**	**7**	**8.45**
	**Temporal_Pole_Sup_L**	**-36**	**15**	**-30**	**11**	**12.54**

**20 TR**	**Temporal_Inf_L**	**-39**	**-33**	**-15**	**7**	**8.72**
	**Frontal_Inf_Orb_L**	**-27**	**21**	**-24**	**7**	**12.46**
	**Rectus_R**	**6**	**27**	**-18**	**13**	**10.25**
	**Insula_L**	**-39**	**-3**	**3**	**7**	**9.73**
	**Insula_R**	**39**	**-3**	**6**	**8**	**10.73**
	**Cerebelum_8_R**	**27**	**-45**	**-48**	**11**	**10.16**
	**Cerebelum_Crus1_R**	**51**	**-57**	**-33**	**8**	**8.59**
**26 TR**	**Temporal_Pole_Sup_L**	**-36**	**15**	**-30**	**7**	**10.81**
	**Rectus_R**	**6**	**27**	**-18**	**9**	**10.06**
	**Insula_R**	**39**	**-9**	**9**	**9**	**11.99**
	**Cerebelum_8_R**	**27**	**-45**	**-48**	**13**	**10.49**
	**Temporal_Inf_L**	**-39**	**0**	**-39**	**6**	**10.64**
**33 TR**	**Temporal_Pole_Sup_L**	**-36**	**15**	**-30**	**6**	**10.01**
	**Hippocampus_L**	**-36**	**-15**	**-15**	**6**	**8.89**
	**Rectus_R**	**6**	**27**	**-18**	**9**	**9.65**
	**Insula_R**	**39**	**-9**	**9**	**8**	**11.73**

Supplementary Material 8: The interaction effects between sleep quality and disease severity by adding GDS as a covariate

Previous study reported that sleep disturbance and depressive symptoms might affect each other. To be specific, whether sleep disturbance may represent early symptoms of AD, or induce cognitive impairment through alterations in sleep-dependent memory

consolidation or through an increase of depressive symptoms is still under debate [19,20]. Here, to explore the possible effect of GDS, we repeated our full factorial analysis by adding GDS as a covariate. To be specific, a 3 × 2 full factorial design, with groups (NC, MCI, and AD) and sleep state (NS and PS) as between-participant factors was performed to explore the interactions between sleep disturbance and disease severity on brain function (dALFF in a whole-brain voxel-wise way). GDS, age, gender, education and FD were employed as covariates. Then, F test was performed by setting the threshold at P<0.001 at a voxel level, with P<0.05 at the cluster level, corrected for multiple comparisons using the Gaussian random field (GRF) method. Results remained largely unchanged (Figure S9, Table S8), suggesting that GDS has no significant effect on the results.

Table S8. The difference of dALFF variance of 6 groups by adding GDS as a covariate

**Neuroimaging Metrics**	**Cluster/peak regions**	**MNI coordinates**			**Extent**	**Max T**
		**X**	**Y**	**Z**		
**dALFF variance**	Cerebelum_Crus1_R	48	-57	-33	8	8.01
	Cerebelum_8_R	24	-45	-45	10	9.31
	Insula_R	39	-3	6	9	10.41
	Insula_L	-39	-3	3	9	10.45
	Temporal_Pole_Sup_L	-36	15	-30	13	14.32
	Hippocampal_R	21	-9	-21	9	10.31
	Rectus_R	6	27	-18	16	10.24
	SupraMarginal_R	57	-42	27	9	10.02

Abbreviation: dALFF, dynamic amplitude of low-frequency fluctuation; GDS, Geriatric

Depression Scale

Figure S8. The difference of dALFF variance of 6 groups by adding GDS as a covariate

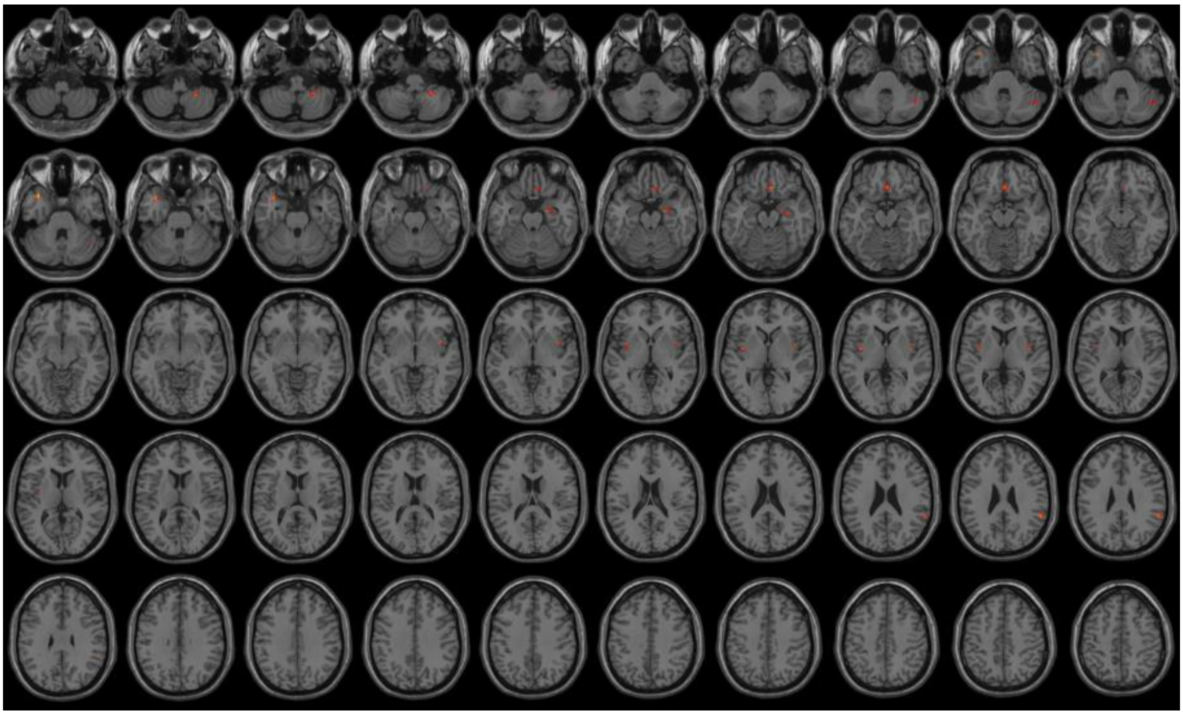

Abbreviation: GDS, Geriatric Depression Scale; dALFF, dynamic amplitude of low-frequency fluctuation

Reference

1 Cummings, J. L. *et al.* The Neuropsychiatric Inventory: comprehensive assessment of psychopathology in dementia. *Neurology* **44**, 2308-2314 (1994).

2 Kaufer, D. I. *et al.* Validation of the NPI-Q, a brief clinical form of the Neuropsychiatric Inventory. *J Neuropsychiatry Clin Neurosci* **12**, 233-239, doi:10.1176/jnp.12.2.233 (2000).

3 Sheikh JI, Y. J. Geriatric Depression Scale (GDS): recent evidence and development of a shorter version. *In: Brink TL, ed. Clinical Gerontology: A Guide to Assessment and Intervention. New York, NY: The Haworth Press;* , 165–173 (1986).

4 Bondi MW, E. E., Jak AJ, Clark LR, DelanoWood L, McDonald CR, Nation DA, Libon DJ, Au R, Galasko D, Salmon DP. Neuropsychological criteria for mild cognitive impairment improves diagnostic precision, biomarker associations, and progression rates. *J Alzheimers Dis* **42**, 275-289 (2014).

5 G. McKhann, D. D., M. Folstein, R. Katzman, D. Price, E.M. Stadlan. Clinical diagnosis of Alzheimer's disease: report of the NINCDS-ADRDA Work Group under the auspices of Department of Health and Human Services Task Force on Alzheimer's Disease. *Neurology* **34**, 939-944 (1984).

6 Mecca, A. P. *et al.* Sleep Disturbance and the Risk of Cognitive Decline or Clinical Conversion in the ADNI Cohort. *Dement Geriatr Cogn Disord* **45**, 232-242, doi:10.1159/000488671 (2018).

7 Moran, M. *et al.* Sleep disturbance in mild to moderate Alzheimer's disease. *Sleep Med* **6**, 347-352, doi:10.1016/j.sleep.2004.12.005 (2005).

8 Benedict, C. *et al.* Self-reported sleep disturbance is associated with Alzheimer's disease risk in men. *Alzheimers Dement* **11**, 1090-1097, doi:10.1016/j.jalz.2014.08.104 (2015).

9 Shi L, C. S., Ma MY, Bao YP, Han Y, Wang YM, Shi J, Vitiello MV, Lu L. Sleep disturbances increase the risk of dementia: A systematic review and meta-analysis. *Sleep Med Rev* **40**, 4-16 (2018).

10 Liguori C, M. N., Nuccetelli M, Izzi F, Bernardini S, Placidi F. Cerebrospinal Fluid Orexin Levels and Nocturnal Sleep Disruption in Alzheimer's Disease Patients Showing Neuropsychiatric Symptoms. *J Alzheimers Dis* **66**, 993-999 (2018).

11 Terum TM, T. I., Rongve A, Aarsland D, Svendsboe E, Andersen JR. The association between specific neuropsychiatric disturbances in people with Alzheimer's disease and dementia with Lewy bodies and carer distress. *Int J Geriatr Psychiatry* **34**, 1421-1428 (2019).

12 Yuen K, R.-R. N., Verhoeff NPLG, Kumar S, Gallagher D, Flint AJ, Herrmann N, Pollock BG, Mulsant BH, Rajji TK, Voineskos AN, Fischer CE, Mah L; PACt-MD Study Group. Association between Sleep Disturbances and Medial Temporal Lobe Volume in Older Adults with Mild Cognitive Impairment Free of Lifetime History of Depression. *J Alzheimers Dis* **69**, 413-421 (2019).

13 Boutin, A. *et al.* Transient synchronization of hippocampo-striato-thalamo-cortical networks during sleep spindle oscillations induces motor memory consolidation. *Neuroimage* **169**, 419-430, doi:10.1016/j.neuroimage.2017.12.066 (2018).

14 Girardeau, G., Inema, I. & Buzsaki, G. Reactivations of emotional memory in the hippocampus-amygdala system during sleep. *Nat Neurosci* **20**, 1634-1642, doi:10.1038/nn.4637 (2017).

15 Alger, S. E., Chambers, A. M., Cunningham, T. & Payne, J. D. The role of sleep in human declarative memory consolidation. *Curr Top Behav Neurosci* **25**, 269-306, doi:10.1007/7854_2014_341 (2015).

16 Sexton, C. E., Storsve, A. B., Walhovd, K. B., Johansen-Berg, H. & Fjell, A. M. Poor sleep quality is associated with increased cortical atrophy in community-dwelling adults. *Neurology* **83**, 967-973, doi:10.1212/WNL.0000000000000774 (2014).

17 Branger, P. *et al.* Relationships between sleep quality and brain volume, metabolism, and amyloid deposition in late adulthood. *Neurobiol Aging* **41**, 107-114, doi:10.1016/j.neurobiolaging.2016.02.009 (2016).

18 Sanchez-Espinosa MP, A. M., Cantero JL. Sleep deficits in mild cognitive impairment are related to increased levels of plasma amyloid-β and cortical thinning. *Neuroimage* **98**, 395-404 (2014).

19 H.A. Keage, S. B., K.L. Yang, K. Morgan, C. Brayne, F.E. Matthews. What sleep characteristics predict cognitive decline in the elderly? *Sleep Med* **13**, 886-892 (2012).

20 Jelicic M, B. H., Ponds RW, Van Boxtel MP, Houx PJ, Jolles J. Subjective sleep problems in later life as predictors of cognitive decline. Report from the Maastricht Ageing Study (MAAS). *Int J Geriatr Psychiatry* **17**, 73-77 (2002).
